# The COVID-19 Pandemic and the Pathology of the Economic and Political Architecture in Cameroon

**DOI:** 10.3390/healthcare8020176

**Published:** 2020-06-17

**Authors:** Nathanael Ojong

**Affiliations:** International Development Studies, 324 Founders College, York University, 4700 Keele Street, Toronto, ON M3J 1P3, Canada; nojong@yorku.ca

**Keywords:** COVID-19, health system, political economy of health, corruption, out-of-pocket payments, Cameroon, structural adjustment, structural violence

## Abstract

This article examines the factors restricting an effective response to the COVID-19 pandemic in Cameroon. It argues that structural adjustment policies in the 1980s and 1990s as well as corruption and limited investment in recent times have severely weakened the country’s health system. This article also emphasises the interconnection between poverty, slums, and COVID-19. This interconnection brings to the fore inequality in Cameroon. Arguably, this inequality could facilitate the spread of COVID-19 in the country. This article draws attention to the political forces shaping the response to the pandemic and contends that in some regions in the country, the lack of an effective response to the pandemic may not necessarily be due to a lack of resources. In so doing, it critiques the COVID-19 orthodoxy that focuses exclusively on the pathology of the disease and advocates “technical” solutions to the pandemic, while ignoring the political and socio-economic forces that shape the fight against the pandemic. At times, medical supplies and other forms of assistance may be available, but structural violence impairs access to these resources. Politics must be brought into the COVID-19 discourse, as it shapes the response to the pandemic.

## 1. Introduction

The current coronavirus disease 2019 (COVID-19) pandemic started in December 2019 [1], and on 31 December 2019, China informed the World Health Organisation (WHO) of numerous cases of pneumonia of unknown cause in Wuhan, a city of 11 million inhabitants [2]. Initially, a significant proportion of those affected worked at the city’s Huanan Seafood Wholesale Market. Three weeks later, there were confirmed cases in the US, Thailand, Nepal, France, Australia, Malaysia, Singapore, South Korea, Vietnam, and Taiwan. The WHO’s Director-General, Dr. Tedros Adhanom Ghebreyesus, declared the novel coronavirus outbreak a public health emergency of international concern on 30 January 2020, after the number of cases increased more than tenfold in a week [3]. By this time, there were confirmed cases in 18 countries, excluding China. On 11 February 2020, the WHO announced a name for the new coronavirus disease: COVID-19. On 11 March 2020, the WHO’s Director-General said that the institution was “deeply concerned both by the alarming levels of spread and severity, and by the alarming levels of inaction”, and concluded that “we have therefore made the assessment that COVID-19 can be characterised as a pandemic” [4]. By this time, there were over 118,000 cases in 114 countries, with 4291 deaths [4]. By 1 May 2020, there were over three million confirmed cases and over 200,000 deaths in 185 countries [5].

In declaring COVID-19 a pandemic, the WHO’s Director-General noted that the “greatest concern is the potential for this virus to spread to countries with weaker health systems which are ill-prepared to deal with it” [4]. Most African countries have weak health systems, including inadequate surveillance and laboratory capacity and limited medical personnel [6]. As of 1 February 2020, only two countries—Senegal and South Africa—had the capacity to test for COVID-19 cases, but by the end of March 2020, over 40 countries had the capacity to do so [7]. The state of the health systems on the continent was captured by the WHO’s Regional Office for Africa: “There is a critical shortage of treatment facilities for critical cases of COVID-19 in Africa […] The total number of beds in intensive care units (ICU) available for use during COVID-19 in 43 countries in Africa is fewer than 5000. This is about five beds per one million people in the reported countries compared to 4000 beds per one million people in Europe […] In 41 countries … functional ventilators in public health services are fewer than 2000.”

As of 18 April 2020, South Sudan, a country of 11 million inhabitants, had four ventilators, and the Central African Republic had three ventilators for its five million inhabitants [8].

The state of the health systems in African countries is of great concern due to the increase in the number of confirmed cases. On 19 March 2020, the WHO reported fewer than 700 confirmed cases in 34 African countries [9], but by May 2020, the Africa Centres for Disease Control and Prevention (Africa CDC), a specialized technical institution of the African Union, recorded 41,330 confirmed cases, 1701 deaths, and 13,621 recoveries. According to the Africa CDC, as of 1 May 2020, Cameroon had the highest number of confirmed cases (i.e., 2069) in the central Africa subregion and the sixth-highest number of confirmed cases on the continent, behind Algeria, Egypt, Morocco, Nigeria, and Ghana [10]. This was a significant increase, as the country recorded its first case on 5 March 2020 [11]. The rapid rise in the number of confirmed cases is also of great concern; as of 1 May 2020, tests had been administered to just 9254 people [10]. There is a consensus among medical professionals that this figure is an underestimation of the number of cases in the country, as testing is limited [12]. According to the Africa CDC, as of 1 May 2020, Ghana had 2169 confirmed cases and had conducted 113,497 tests. Certainly, the number of confirmed cases in Cameroon will be much higher if more tests are conducted. The outbreak and its spread pose an additional challenge for Cameroon due to the armed conflict in the Far North, Northwest, and Southwest regions. UNICEF notes that 34% of health facilities in the Northwest and Southwest regions are non-functional or only partially functional (absent health personnel, destroyed infrastructure, and lack of medical supplies), and access to health care is limited [13].

The occurrence of COVID-19 and past deadly coronaviruses, together with the mode of spread of these viruses as outlined below, are not contested. The focus has been on the epidemiology of COVID-19 [14,15,16,17] and weak health systems [6]. The mobilising narrative has been around health security [18,19], and thus emphasis has been put on public health measures to prevent, detect, verify, assess, and respond to the pandemic, as stipulated by international health regulations [20]. At present, existing scholarship has dedicated very little attention to the local political, economic, and social factors that shape the fight against COVID-19, which is surprising, as scholars have highlighted the political economy of recent epidemics, including COVID-19 [21,22,23,24,25,26,27]. Sanders and colleagues, for example, stressed the economic and political forces that have contributed to the severe weakness of health systems of West African countries which were seriously affected by the Ebola virus [23], and COVID-19 highlights the need to take inequality and social stratification in Africa seriously [28]. This paper adds to this emerging research stream by analysing the political economy of the COVID-19 pandemic in Cameroon and addressing two core questions: (a) What are the economic and political roots of Cameroon’s weak health care system? and (b) What are the factors restricting an effective response to COVID-19 in Cameroon?

This paper proceeds as follows: first, I examine how COVID-19 spreads, and the next section presents an analysis of the economic and political roots of Cameroon’s weak health care system. This is followed by a discussion of the factors restricting an effective response to COVID-19 in the country, while the subsequent section shows that the issues experienced in Cameroon are similar to those in neighbouring countries. I conclude by highlighting the main arguments.

## 2. Methods and Data

This review draws on secondary data sources, and the evidence presented is based first on the growing body of literature regarding the COVID-19 pandemic from a variety of institutional fora. A significant proportion of this work has been carried out by people directly or indirectly associated with international institutions (e.g., the World Health Organisation and World Bank), academic institutions, news agencies, national government departments, and non-governmental organisations (NGOs). In some cases, data from these sources were readily available online. This paper relies heavily on scholarly and national government literature to historicize the state of the health sector, while some of the evidence supporting the analysis is drawn from local and international news agencies. The evidence provided by these news agencies is current, which explains why they are used to shed light on contemporary issues related to health care, including COVID-19. Additionally, news agencies often bring to the attention of the public issues that governments are not ready to disclose for political reasons, and scholars have endorsed the use of these sources [29,30].

The use of these secondary sources poses some limitations, as local new agencies may be biased regarding reportage [30]. To minimise this problem, evidence is drawn from a variety of national and international news agencies. Additionally, the statistics provided by national government agencies should be considered with caution, as across multiple African countries, there are discrepancies between administrative data and independent household surveys [31]. Several African countries also misreport to foreign donors [31]. So, these limitations should be taken into consideration when engaging with discussions in this paper. That said, the goal of this review is to provide a deeper understanding of the factors that have contributed to weakening Cameroon’s health sector over the years and to shed light on socio-economic and political factors that are currently restricting an effective response to the pandemic in the country.

## 3. How COVID-19 Spreads

Coronaviruses are pathogens that primarily target the respiratory system in humans [32]. The most common symptoms at the onset of COVID-19 are fever, cough, and fatigue, while other symptoms include headache, sputum production, hemoptysis, dyspnoea, diarrhoea, and lymphopenia [33,34,35]. The incubation period for COVID-19, i.e., the time between exposure to the virus and symptom onset, is on average 5–6 days, but can be up to 14 days [36]. However, some people are asymptomatic, meaning that they are infected with COVID-19 but do not develop symptoms.

Scholars have suggested that a “wet market” in Wuhan city where live animals are often sold may be the zoonotic origin of COVID-19 [32]. The WHO has classified COVID-19 as a β-coronavirus of group 2B [37], with a genome that is highly similar to bat coronavirus, thus pointing to bats as the natural host [1,38]. Research has shown that some bat SARSr-CoVs have the potential to infect humans [39,40]. It seems that most of the early confirmed cases had a contact history with the original wet market in Wuhan [1]; however, COVID-19 is currently being transmitted by human-to-human contact. COVID-19 uses the same receptor, angiotensin-converting enzyme 2 (ACE2), as that for SARS-CoV, and spreads principally through the respiratory tract [38]. Human-to-human transmission occurs primarily through direct contact or through droplets spread from an infected person by coughing or sneezing [32]. Human-to-human transmission has accounted for the proliferation of COVID-19 across the globe.

## 4. Economic and Political Roots of Cameroon’s Weak Health Care System

A strong health care system is vital in handling confirmed cases and reducing the COVID-19 fatality rate. As was mentioned earlier, Cameroon, like other African countries, has a weak health care system. So, what is the state of Cameroon’s health system, and what factors have contributed to weakening it?

### 4.1. The State of Cameroon’s Health System

The public health sector is considered one of the driving forces of Cameroon’s health care system due to its core objectives of disease prevention and providing and improving health services to its population. Public health facilities are organised into seven categories: general hospitals, central hospitals, regional hospitals, district hospitals, district medical centres, integrated health centres, and ambulatory health centres [41]. In addition to these seven groups, there are also private clinics, health facilities operated by religious organisations and NGOs, and traditional health institutions. Quantitatively, in 2014, there were 4034 public and private health facilities in the country (Table 1)—of which, 27.9% were private institutions (i.e., for-profit or non-profit institutions) [42]. Some public health institutions are not fully functional due to lack of equipment, and where equipment exists, it is obsolete or of poor quality [42]. One study identified a lack of delivery kits (24.5%), dry-heat sterilization systems (39.5%), Caesarean section kits (67.5%), and functional microscopes (11.6%) in health institutions in the country [43]. The ratio of health personnel (medical doctors, midwives, and nurses) to the regular population is 1.07 per 1000 inhabitants [42,44]. More precisely, there are 0.8 doctors per 10,000 people, and 13 hospital beds per 10,000 people [45]. In some administrative regions, the proportion is as low as 0.3 doctors per 10,000 people (Table 1). The government admits that public health institutions are understaffed [42].

Public resources allocated to the health sector in Cameroon remain some of the lowest in Africa in terms of GDP [47,48]. Out of the US$61 per Cameroonian spent on health care in 2010, the government contributed only US$17, i.e., 28%—of which, US$8 was provided by international donors [47]. Therefore, the cost of health care is largely borne by individuals through out-of-pocket payments. In 2015, out of US$156 per person spent on health care in the country, US$108 was out-of-pocket spending, US$23 was government spending, US$20 was development assistance for health, and US$5 was prepaid private spending [49]. The national and international press have reported cases in cities such as Douala and Yaoundé where people were denied health care or detained because of their inability to pay their health care bills. In 2012, the BBC reported the case of a mother and baby who were detained for 11 months by a hospital in Yaoundé due to the mother’s inability to pay her medical bill [50], and in 2018, the France-based news agency France24 reported that a hospital in the capital city, Yaoundé, detained ‘about a dozen mothers and their newborns in a small room for about a month because they were unable to pay the hospital fees for the birth by Caesarean section’ [51].

People have also had to rely on out-of-pocket payments to cover health care costs related to COVID-19. An independent local news agency in the country reported that some public health institutions in Douala required COVID-19 patients to cover their health care costs. The news agency interviewed the spouse of a COVID-19 patient who, after spending approximately 200,000FCFA ($332) on tests and prescription drugs, turned to the use of free herbal medicine provided by the Archbishop of the Douala Metropolitan Archdiocese, His Lordship Samuel Kleda [52]. The country’s Minister of Public Health, Dr. Malachie Manaouda, in a press release published on 16 April 2020, declared a ban on the systematic billing for screening tests, hospitalisations, and administration of prescription drugs. However, two weeks after the minister’s press statement, people in Douala, for example, were still relying on out-of-pocket payments for COVID-19-related medical expenses [53]. The confusion surrounding the requirement of COVID-19 patients to cover their medical bills is captured in the following statement by the Director of the Douala Gyneco-Obstetric Hospital, Prof. Emile Mboudou: “Is it possible that the hospital gives you a prescription and also gives you the money to buy the prescribed drugs in a pharmacy? We have not received a drug endowment until now at the Douala Gyneco-Obstetric Hospital” [53]. This case depicts a lack of coordination between the ministries of public health and finance. The former has the technical authority to instruct medical facilities not to charge COVID-19 patients, but for this policy to take effect, the latter must make the funds available to the medical institutions to cover the costs.

The COVID-19 pandemic has made manifest Cameroon’s weak health system. As of 10 April 2020, the country had just four testing laboratories, with three of them in the capital Yaoundé [12]. A medical doctor in Douala, the country’s economic capital, noted: “There are less than 10 ventilators in the whole city. We are having challenges in treating patients with acute respiratory distress” [12]. The country’s prime minister, Joseph Dion Ngute, announced plans to transform eight venues into makeshift medical facilities to be used for the treatment and follow-up of COVID-19 patients, but the construction of these makeshift medical facilities, as well as equipping them, has been slow [54]. As of 30 April 2020, none of these makeshift medical facilities was ready to receive COVID-19 cases.

Cameroon has received external support to fight COVID-19. The country received medical supplies from UNICEF and Jack Ma’s Alibaba Foundation [55], 14 vehicles from the WHO, and financial support from countries such as the United States of America [56] and Switzerland [57]. The NGO Doctors Without Borders (Médecins Sans Frontières) has also been supporting the country’s response to the COVID-19 pandemic. Although the humanitarian assistance provided is laudable and timely, it reinforces structures of dependence, and history tells us that the country is likely to continue to depend on external assistance, including foreign experts to tackle future epidemics. Scholars have argued that sub-Saharan African countries which receive aid are less likely to have incentives to invest in effective public institutions [58]. Therefore, the government may not have the incentives to invest in the health care system, as it is well aware that it can always count on external support in times of emergency.

Undoubtedly, COVID-19 has exposed Cameroon’s weak health care system, and to understand the current state of the country’s health system, it is important to investigate its roots. Put simply, it is important to examine the factors that have contributed to weakening the country’s health system.

### 4.2. Structural and Socio-Economic Factors

Cameroon’s weak health system can be traced to the years of structural adjustment which began in the mid-1980s. After independence, Cameroon enjoyed relative economic prosperity until the mid-1980s. From 1970 to 1985, its economy grew annually at approximately 8% [59], which led US President Ronald Reagan to refer to the country as a ‘shining example for Africa’ [60]. This growth was mostly due to the boom in exports of cash crops. In 1977, cash crops made up 71.9% of exports, while oil comprised only 1.4% [59]. The structure of the country’s economy changed in the 1980s because of oil exploration. In 1985, oil made up 65.4% of exports and cash crops 21.4%, with the government receiving high royalties from international oil companies developing the field [59]. According to the World Development Indicators database, GDP per capita (in 2010 US$) increased from $1122.2 in 1975 to $1688.9 in 1984. However, Cameroon’s impressive economic performance was short lived, and in 1985/1986, a drop-in oil revenue due to a simultaneous reduction in prices and exploitable sites and a decline in the terms of trade for cash crop exports slowed down the economy’s growth [61]. Farm prices for cocoa, Arabica and Robusta coffee, rice, and cotton declined by 45, 47, 60, 44, and 32%, respectively [62]. Accompanying the sluggish economy were budget and balance-of-payment deficits, a build-up of internal arrears, a rise in foreign indebtedness, and worsening solvency problems for commercial banks [62].

External borrowing and reserves held abroad permitted the government to push back any sort of reform until 1987, when President Paul Biya announced some budget cuts [63]. However, this measure failed to remedy the situation, and deficits continued to rise. Between 1985 and 1992, real GDP fell by 30%, the external deficit averaged 6% of GDP, foreign debts tripled to over 60% of GDP, and the debt-service ratio increased to 42% [61]. By 1988, the government had no option but to enter into a structural adjustment agreement with the World Bank [60].

#### 4.2.1. Effects of Structural Adjustment Policies

Structural adjustment policies in Cameroon included devaluing the currency, cutting public expenditures, eliminating subsidies, promoting exports, especially agricultural, and liberalising trade [64]. Pay cuts were introduced in 1991 (7%) and 1993 (30% at first, and later 50%) [59]. Prior to the pay cuts in 1993, an integrated public employee on index 1140 earned a gross salary of 420,425 FCFA (US$714), and after the cuts, their salary decreased to 154,287 FCFA (US$262), i.e., a 63.3% reduction in pay [65,66]. Then, in 1994, there was a 50% devaluation of the FCFA in return for $114 million in credit ratified by the International Monetary Fund [59]. Additionally, as of 1989, public service employees began to experience delays in salary payments which usually exceeded three months [67]. According to the World Development Indicators database, per capita GDP (in 2010 US$) decreased from $1688.9 in 1984 to $1047.2 in 1994.

The World Bank- and IMF-sponsored structural adjustment programmes severely affected the public health sector in the country, and there was no recruitment of people into the public health sector for 15 years [68]. Further, there was little investment in health infrastructure [69]. Paramedical training for laboratory technicians and nurses was suspended for several years, and training schools closed [68]. Low salaries and poor working conditions [69] led public health personnel to move to the private health sector where salaries were higher [68], or to move abroad [70]. In 1999, public health sector jobs were approximately 80% unfilled [68].

Unsurprisingly, the structural adjustment policies also directly affected users of public health institutions. Government spending on public services was curtailed, and “health became a commodity and an individual responsibility” [26]. The government implemented a health cost recovery system that required users of public health institutions to cover their health care costs, and this implementation of out-of-pocket payments amid chronic poverty pushed people to turn to alternatives such as self-medication, traditional medicine, and drugs from street vendors [71]. Drugs purchased on the street are often counterfeit or substandard, causing harm to patients as well as failing to treat the diseases for which they are intended. People’s decisions to purchase counterfeit or substandard drugs on the street were not solely due to the cost of these products in formal health care institutions. The government’s reduction in health spending meant that some public health institutions did not have the medications required by patients, leading the latter to purchase drugs on the street [72]. Unlike the public health institutions, people did not have to travel long distances to purchase counterfeit or substandard drugs, as these were readily available in local communities. Put simply, availability and accessibility were and remain key contributory factors to the proliferation of counterfeit or substandard drugs in the country [72].

#### 4.2.2. Chronic Corruption

The shortage of drugs in public health institutions was also linked to chronic corruption, which remains pervasive in the country. Cameroon was ranked the most corrupt country in 1998 (out of 85 countries surveyed) and 1999 (out of 99 countries surveyed), and in 2019 it ranked 153rd out of 180 countries in the 2019 Corruption Perception Index. Cameroon’s score of 25 out of 100 indicates serious levels of public sector corruption [73]. Regarding judicial independence, in 2019, the country had a value of 2.9 out of 7, indicating that its judicial system is seriously influenced by members of the government as well as private citizens and firms [74]. In the past, there has been large-scale drug thefts and small-scale pilfering, and the limited drugs that reached public health care institutions due to the reduction in public health expenditures were given to local authorities, family members, and friends before patients could benefit from them [75]. It is no coincidence that data from the 2011 household survey conducted by the government show that approximately 85% of household heads in urban and semi-urban areas noted the high level of corruption in the public health sector [76]. A study conducted in the city of Douala found that in order to avoid long wait times to see a doctor, people offered bribes in order to enable them to skip the queue [77]. Additionally, some doctors in public hospitals operate private clinics, so when patients go for consultation in public hospitals, these doctors often direct them to their own clinics, where costs are significantly higher [77], and this practice also increases the total health care costs borne by patients.

Funds provided by external partners, intended to help the government strengthen the country’s health system, have been misused too. This was the case for funds provided by the GAVI Alliance, a public–private partnership whose mission is to save children’s lives and protect people’s health by increasing access to immunisation in the Global South. The GAVI Alliance uncovered massive misuse of its grants in Cameroon in 2011, and an investigation led by the alliance revealed that “of US$5.1 million programme expenditures, US$3.7 million had been misspent, partly due to fraud. Different types of corruption affected this programme” [78]. The investigation highlighted fraud in purchasing (e.g., non-existent suppliers, order-splitting to avoid tender, fake invoices, over-invoicing of 900–1600% higher than market prices, purchase of incompatible supplies, unjustified repairs), fraud in activities (e.g., funding of fictitious activities, funding of activities already funded by other partners, withdrawals for activities not undertaken, payment of unauthorized per diems), and unjustified cash disbursements (e.g., discrepancies between bank withdrawals and the amounts in supporting documentation). In fact, a business address on one of the fake invoices was found to actually be a cemetery, and ‘brand new vehicles were allegedly subjected to “repairs” costing thousands of dollars’ [78].

Several top government officials have been imprisoned for corruption. For example, in 2015, a former finance and economy minister was sentenced to 25 years in prison for embezzling US$11 million [79]. However, some political analysts contend that the arrest and imprisonment of some high-profile government officials, allegedly for embezzlement of funds, is politically motivated [80]. The government claims that the country has lost approximately US$1.5 billion in stolen funds [80], but corruption is just one of several factors which have contributed to weakening the country’s health system.

#### 4.2.3. Limited Public Investment in the Health Sector

**Figure 1:**
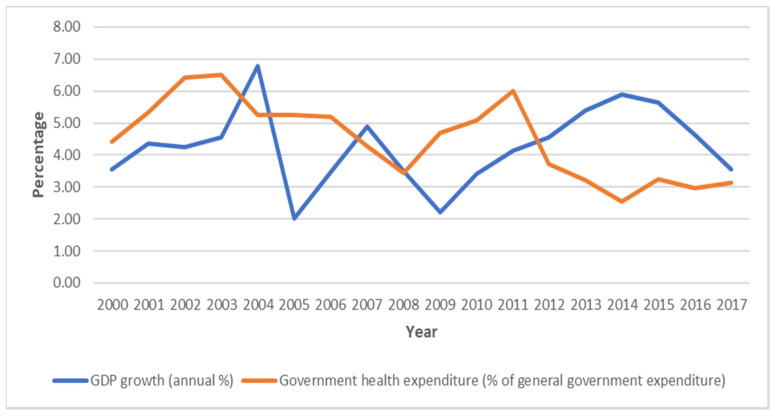
GDP growth and government health expenditures in the period 2000–2017. Source: author’s computation based on data from World Development Indicators database.

Deepening the problems is the limited state investment in health care, and even after the country entered a period of successive GDP growth, there was no significant increase in health spending as a percentage of government expenditures (Figure 1). Instead of an increase in public health spending as a percentage of general government expenditures, there has been a decrease; e.g., between 2011 and 2017, the country recorded an annual GDP growth rate of at least 3%, but there was a decrease in health expenditure as a percentage of general government spending from 6% in 2011 to 3.2% in 2015, and then to 3.1% in 2017 (Figure 1). So, the weak health care system is also due to years of inadequate investment in the public health sector. The point emphasised here and elsewhere is that state institutions shape the response to COVID-19, and put differently, this trend is indicative of how structural violence hampers the fight against the disease. By structural violence, I refer to the ‘way institutions and practices inflict avoidable harm by impairing basic human needs’ [26]. Apart from the weak health care system, however, there are several other factors restricting an effective response to the COVID-19 pandemic.

## 5. Factors Restricting an Effective Response to the COVID-19 Pandemic

So far, I have examined the factors which have contributed to weakening Cameroon’s health care system. As the fight against the COVID-19 pandemic requires bringing together various vital elements, this section focuses on the socio-economic and political factors currently restricting an effective response to the pandemic.

### 5.1. Poverty, the Informal Economy and Lack of Basic Amenities

In addition to the weak health care system, other factors such as poverty and a lack of basic amenities are hindering the fight against COVID-19. According to the country’s National Institute of Statistics, approximately 37.5% of the population lives below the national poverty line of 931 FCFA (US$1.5) per day [81]. The World Bank noted that between 2007 and 2014, the number of poor people increased by 12%, to approximately 8 million. However, these figures should be considered with caution, as there is a consensus among researchers that official statistics in several sub-Saharan African (SSA) countries are inadequate and unreliable [31,82]—what Shantayanan Devarajan refers to as the “statistical strategy” [83]. Unemployment among those aged 15 to 35 is approximately 13%, but crucially, underemployment is 71.9% at the national level and 54.4% and 79.2% in urban and rural zones, respectively [84], while informal employment stands at 88.6% [85].

The precariousness of everyday life makes it challenging to prevent the spread of COVID-19. On 17 March 2020, the government announced restrictive measures, including the closure of land, air, and sea borders, closure of schools, a ban on gatherings of more than 50 people, and a restriction of non-essential urban and interurban travel within the country. From the beginning, however, it was clear that it would be difficult to implement these measures. It is challenging to ensure physical distancing and the flow of people in marketplaces which are often overcrowded, especially when a significant proportion of the population is not cooperative. Moreover, as earlier mentioned, over 80% of the population relies on the informal economy for a living, and since the functioning of the informal economy is based on the movement of people, without an outright lockdown, it is almost impossible to prevent the interurban travel of informal workers. As well, the government may not be willing to impose a lockdown since over 80% of the working population have livelihoods in the informal economy. Additionally, the government made it obligatory from April 13 for people to wear face masks whenever appearing in public as part of measures to slow the spread of the disease, but due to the high poverty rate in the country, most people say they cannot afford a mask.

A lack of basic amenities is also affecting the fight against the pandemic. Frequent power outages in the country render it difficult for people who have food preservation appliances to make use of them, and for low-income populations who cannot afford a power generator, this means they must go out on a regular basis to purchase fresh food. Water shortages also plague major cities in the country. The over three million residents in the country’s capital, Yaoundé, require a daily supply of approximately 300,000 cubic meters of clean water, but only 35% of this is provided [86]. It is tough persuading someone staying in a one-room unit with family members, without water and power, to stay indoors as much as possible and only go out when necessary.

Additionally, the closure of schools has simply moved most primary and secondary school students from low-income households from the classroom to the streets. In order to make ends meet in the household, some parents send their children to sell goods in the streets and marketplaces. One student told a local news agency that “we are afraid to contract COVID-19 but we have to sell; we won’t have food to eat if we stay home” [87]. Another said that since the schools closed, she had been helping her mother sell goods in the market, adding that they would not have food to eat if they did not perform these activities [87]. As those who make a living in the informal economy have no income if they do not work, they are forced to continue their activities even at the risk of contracting the disease.

Crucially, the discussion regarding poverty shows how it causes the spread of COVID-19, as low-income populations who work in the informal economy are forced to go outside in order to gain income, and as they often work in crowded environments, this exposes them to the virus. When they get the virus, it is likely to spread faster within their households, as they are often overcrowded. Different households in the slums often share toilets and bathrooms [62], thus facilitating the spread of the virus from one household to another. Scholars have documented the complex interconnections between poverty, slums, and disease in Africa [27,88].

The interconnections between poverty, slums, and COVID-19 bring to the fore the social inequality in Cameroonian society. Pandemics rarely affect populations in a uniform way [89], and arguably, the experiences with COVID-19, i.e., the nature of the infection, the rate of spread, and access to medical care, vary according to class. As mentioned earlier, low-income populations are more likely to get infected; the upper class can self-impose lockdowns, as they have food, power generators, access to potable water and the internet, and their households are not overcrowded. Their status in everyday life reduces their rates of infection, and those who get infected can afford quality medical care. I do not suggest, however, that the better-off populations constitute a homogenous class. The point emphasised here is the different impacts of COVID-19 on different populations. Arguably, if low-income populations are more likely to get infected and spread it to others in their households and communities, one might argue that inequality may facilitate the spread of COVID-19 in the country. Scholars have put forward points that seem to support the relationship between inequality and the spread of COVID-19 [89].

### 5.2. Weak Law Enforcement Mechanisms

In addition to the issues of poverty and social inequality, weak enforcement mechanisms also restrict effective responses to the pandemic. To prevent the spread of the disease, the government recommended that travellers coming into the country be quarantined for 14 days, and travellers arriving at international airports in Douala and Yaoundé were taken to hotels in these cities for quarantine. However, some of these people received visits in their hotel rooms from family members, friends, and even prostitutes. A top government official in the administrative division of Mfoundi, where Yaoundé is located, angrily noted, “We discovered that people put in quarantine were conniving with hotel agents [workers] to smuggle women into the hotel to sleep with them. We have arrested some of them. We have to work together to stop this virus” [90]. The official added that he ordered the arrest of 50 prostitutes as well as 13 women and six men who had sneaked into hotels to meet their spouses [90]. Moreover, 150 of those quarantined escaped from their hotels, and 186 people who returned to the country from France and Italy refused to be isolated [90]. In Cameroon, the locals often say that “money speaks”—in other words, people in a sound financial position can make things happen. Most of those who escaped were people who had returned from Western countries such as France, Italy and Belgium, and their social class may have made it easier for them to disregard the guidelines. The violators seem to have exploited the country’s weak enforcement mechanisms, as people tend to get different treatment based on their social class. The WHO has urged national governments to “find, isolate, test and treat every case and trace every contact” [4], but it is challenging to implement this protocol in an environment where corruption is pervasive and where there are weak law enforcement mechanisms.

### 5.3. Political Factors

The current political climate in Cameroon is affecting the fight against the COVID-19 pandemic there. As mentioned earlier, there is armed conflict in the Far North, Northwest, and Southwest regions. What began in 2017 as a political crisis linked to discrimination against English-speaking regions in the country, i.e., the Northwest and Southwest regions, became a deteriorating humanitarian emergency. The United Nations has noted that the conflict in the Northwest and Southwest regions has created a humanitarian emergency affecting approximately 1.9 million people [91]. As of August 2019, there were 450,268 internally displaced persons in the Northwest and Southwest regions and 270,870 internally displaced persons in the Far North region [13].

For political reasons, the government has repeatedly downplayed the severity of the displacement and the humanitarian need, putting it at odds with aid agencies, including the United Nations office in the country. For example, in 2019, the government, through the Minister of Foreign Affairs, said that aid agencies had inflated the number of internally displaced persons in order to receive aid from donors, and noted that only 150,000 displaced families have been identified and that the government was already providing humanitarian assistance to 75,000 of them [92]. The government has focused on blocking the delivery of aid to the Northwest and Southwest regions to show that there is no humanitarian crisis in these regions [93], and the government recently suspended flights by aid groups to these regions [12]. Officially, the government claims that this is to prevent the spread of COVID-19, but the move may have more to do with politics, and seems to be part of a government strategy to restrict aid agencies’ access to these regions in order to in turn prevent access to information on the ground and thus impose its narrative. There are confirmed cases in both regions, so by suspending the UN Humanitarian Air Service, the government is preventing aid, including medical supplies, from reaching the most vulnerable people.

In a news conference, the WHO’s Director-General famously said, “Do not politicise this virus.” It seems that his call was not heeded in Cameroon. On 3 April 2020, opposition leader Prof. Maurice Kamto launched the “Survie-Cameroon-Survival Initiative” (SCSI) in order to raise funds to fight COVID-19 in the country. In response, the country’s Minister of Territorial Administration said that any appeal to public generosity, for whatever reason, must be authorized by his ministry, considering the SCSI to be “illegal”, and ordered banks and mobile phone operators to close accounts linked to SCSI. To the government, the launch of SCSI was a direct challenge to the solidarity fund set up by the government to fight COVID-19. On 30 April 2020, a gift of approximately 17,000 face masks and 950 test kits from Prof. Maurice Kamto under the banner of SCSI was rejected by the Minister of Public Health, and the SCSI coordinator was told to take the gift to the Ministry of Territorial Administration [94]. According to the government, SCSI was functioning illegally, as it had not received authorization from the Ministry of Territorial Administration to collect public donations. It is based on this narrative of illegality that the Minister of Public Health said that the gift would have been received if the opposition leader had presented it as an individual and not through SCSI, concluding that “just because we are in an epidemic does not mean that we have to set aside our laws and regulations … this must be emphasized. We did not refuse to do so [receive the gift], but we simply asked him to get in touch with the Ministry of Territorial Administration which oversees associations” [94]. Based on the aforementioned points, it is clear that some people are not receiving assistance because of politics. On 11 May 2020, six volunteers from SCSI were arrested while handing out free protective masks and sanitizing gel to residents of Yaoundé, the capital [95]. Some medical facilities are in need of vital supplies, low-income people are in need of masks and hand sanitizer, and communities affected by conflict lack food and other basic necessities, while politicians seem to be focused on scoring political points. This is another manifestation of structural violence.

## 6. Beyond Cameroon: The Case of Neighbouring Countries

Although the focus here has been on Cameroon, some of the core issues discussed so far are not unique to the country. Corruption is also pervasive in neighbouring countries such as Gabon, Nigeria, Central African Republic, Chad, Republic of Congo, and Equatorial Guinea. According to the 2019 Corruption Perception Index, these countries occupied the 123rd, 146th, 153rd, 162nd, 165th, and 173rd positions out of 198 countries, respectively, thus indicating very high levels of corruption [73]. Based on these rankings, these countries are worse off compared to other African countries such as Botswana (which ranked 34), Rwanda (ranked 51), and Mauritius (ranked 56). In comparison, Nigeria and Chad were the most corrupt countries in 1997 and 2005, with rankings of 99 and 100, respectively [96,97].

Similar to Cameroon, investment in the public health system has also been limited in neighbouring countries. Based on data from the World Bank’s World Development Indicators and Stockholm International Peace Research Institute (SIPRI), in Chad, for example, while military spending increased when the country started receiving oil revenues in 2003, except for 2004 and 2005, there has been a decrease in health expenditures as a percentage of government spending. According to data from the World Bank’s World Development Indicators, health expenditures in Chad as a percentage of government spending decreased from 11.3% in 2000 to 7.1% in 2006, and then to 3.4% in 2010. In the Republic of Congo, another oil-exporting country, data from the World Bank’s World Development Indicators show that since 2000, health expenditures as a percentage of general government expenditure have been below 5%. Unsurprisingly, these countries have weak health care systems. The health system is even worse in the Central African Republic, which has been ravaged by a protracted civil war. As of April 2020, the UN’s Office for the Coordination of Humanitarian Affairs (OCHA) noted that there were only three ventilation kits, one oxygen concentrator, and one COVID-19 treatment centre with 14 beds in that country [98].

As in Cameroon, the pandemic has been politicised in neighbouring countries. The government of Equatorial Guinea recently expelled the country’s WHO representative based on a claim that the representative had falsified the country’s tally of COVID-19 cases [99], since figures published by the WHO have sometimes been higher than those put forward by the country’s government. Additionally, Equatorial Guinea’s official tally of COVID-19 cases had been being updated daily, but the practice ceased on 28 April 2020 [100]. This case corroborates the point mentioned earlier regarding the political economy of data in African countries.

The point emphasised here is that Cameroon’s economic, social, and political issues are not unique to that country. Although there are differences in terms of specifics, there is a general pattern noticeable in Cameroon and its neighbour countries, and moreover, COVID-19 has equally exposed the weak health care systems in those countries.

## 7. Conclusions

The COVID-19 pandemic has exposed weak health systems in several countries, especially those in the Global South. Medical experts are currently focused on the epidemiology of the disease, and rightly so, due to its high fatality rate, as the disease has so far claimed the lives of over 400,000 people. So, scientists are racing to develop a vaccine, and in the meantime governments around the world have implemented restrictive measures aimed at containing the spread of the disease. Although these efforts are laudable, I argue that it is important to examine the political economy of COVID-19, as political and economic forces influence the fight against the disease.

Using Cameroon as a case study, I have examined the economic, political, and social forces that negatively affect the fight against COVID-19, and argue that the country’s weak health care system makes it challenging to tackle the disease there as well as in other countries. A combination of structural adjustment policies in the 1980s and 1990s as well as corruption and limited investment in recent times have severely weakened the country’s health system, causing poor and vulnerable populations to suffer the most. Additionally, politicians are using the pandemic to score political points, as, for political reasons, the government has prevented aid, including medical supplies, from humanitarian organisations from reaching vulnerable populations in certain regions. Based on the foregoing, I contend that the inability to tackle the COVID-19 disease may not always be due to a lack of medical supplies or other forms of assistance. As I have shown, aid is at times available, but some people are not able to access it. Put differently, political forces are thwarting the response to COVID-19 in Cameroon, so politics must be brought into the discourse. The response to COVID-19 in Cameroon is a political process, and strategies produced by various actors in the development community cannot be effective if the complexity of local politics is not taken seriously.

It is also worth noting that the pandemic has also brought to the fore the weaknesses of health-systems in Western countries, as several countries in the West have also been facing challenges in tackling the pandemic due to years of budget cuts that have weakened their health care systems. The major difference between African countries and Western countries is that in most cases, the latter have the capacity to mobilise resources needed by health care systems at short notice, while the former often do not have that capacity.

## Figures and Tables

**Table 1:** Distribution of health facilities per region in Cameroon.

Administrative Regions	Population (2012)	No. of Health Institutions (2014)	No. of Doctors per 10,000 People (2011)	No. of Nurses per 10,000 People (2011)
Adamawa	1,098,165	157	0.4	8.8
Centre	3,803,931	838	2	14.6
East	818,139	227	0.7	11.3
Far North	3,709,691	329	0.3	5
Littoral	3,085,304	619	1.5	13.1
North	2,240,649	272	0.4	8.1
North-West	1,870,148	367	0.3	4.7
West	1,840,137	628	0.8	12.8
South	720,833	310	0.8	11.4
South-West	1,449,957	287	0.6	10.9
Total	20,636,954	4034		

Source: Adapted from Ministry of Health 2017; Ministry of Public Health 2012 [41,46]. Note: the statistics for number of doctors per 10,000 people and number of health institutions per administrative region are the most recent available.
